# Characterization and classification of Romanian acacia honey based on its physicochemical parameters and chemometrics

**DOI:** 10.1038/s41598-020-77685-9

**Published:** 2020-11-26

**Authors:** Mihaela Emanuela Crăciun, Oana Cristina Pârvulescu, Andreea Cristina Donise, Tănase Dobre, Dumitru Radu Stanciu

**Affiliations:** 1 Department of Analytical Chemistry and Environmental Engineering, University POLITEHNICA of Bucharest, 1-6 Gheorghe Polizu, 011061 Bucharest, Romania; 2 Department of Chemical and Biochemical Engineering, University POLITEHNICA of Bucharest, 1-6 Gheorghe Polizu, 011061 Bucharest, Romania; 3 Department of Economic Engineering, University POLITEHNICA of Bucharest, 313 Splaiul Independentei, 060042 Bucharest, Romania

**Keywords:** Chemistry, Engineering

## Abstract

Three groups of Romanian acacia honey, i.e., pure, directly adulterated (by mixing the pure honey with three sugar syrups), and indirectly adulterated (by feeding the bees with the same syrups), were characterized and discriminated based on their physicochemical parameters. Moisture, ash, 5-hydroxymethylfurfural (HMF), reducing sugars (fructose and glucose), and sucrose contents, free acidity, diastase activity, ratio between stable carbon isotopes of honey and its proteins (*δ*^13^*C*_*H*_ and *δ*^13^*C*_*P*_) were evaluated. Adulteration led to a significant increase in sucrose content, HMF level, and Δ*δ*^13^*C* = *δ*^13^*C*_*H*_‒*δ*^13^*C*_*P*_ as well a decrease in reducing sugar content and diastase activity. Principal component analysis (PCA) and linear discriminant analysis (LDA) were applied to experimental data in order to distinguish between pure and adulterated honey. The most relevant discriminative parameters were diastase activity, HMF, sucrose, and reducing sugar contents. Posterior classification probabilities and classification functions obtained by LDA revealed that 100% of honey samples were correctly assigned to their original group.

## Introduction

Honey is a natural foodstuff produced by honey bees from flower nectar (blossom honey) as well as from the secretions of all plant parts other than flowers or excretions of sap-sucking insects on the plants (honeydew honey)^1^. It consists of sugars (70–85%), water (10–20%), and low amounts of other compounds, including proteins (especially enzymes), amino acids, organic acids, phenolic compounds, vitamins, and minerals^2–16^. Monosaccharides (fructose and glucose) represent about 75–95% of sugars, disaccharides (sucrose, maltose, isomaltose, turanose, nigerose, trehalose) about 10–15%, trisaccharides and higher sugars (maltotriose, dextrins, raffinose) up to 2%^2,3,5,13,15,17–20^. The bees convert disaccharides and trisaccharides from nectar into monosaccharides, e.g., sucrose into glucose and fructose (in the presence of invertase), maltose and maltotriose into glucose^3,5^. Moreover, glucose can be converted into gluconic acid and hydrogen peroxide in the presence of glucose oxidase enzyme^3,5,13^. Honey composition and properties can be heavily influenced by botanical and geographical origin, climate, season, harvesting time, processing and storage conditions. Due to its high price and limited availability, the honey is often subjected to adulteration. It can be made either directly, usually by mixing with different inexpensive sugar syrups (of sugar cane or beet, corn, rice, date, agave, inulin, inverted sugar, glucose, fructose), or indirectly, by feeding the bees with various sugars^2,5,7,10,13–16,19–31^. The demand for authentic and high quality honey has recently increased. In this context, finding the most effective methods to detect honey adulteration is an imperative issue.

Traditionally, honey adulteration is detected by measurements of relevant physicochemical parameters [e.g., moisture, ash, proline, 5-hydroxymethylfurfural (HMF), reducing sugar (RS) and non-reducing sugar (NRS) contents, electrical conductivity, diastase activity (DA), free acidity (FA), pH, rheological parameters]. These targeted analytical techniques, which are generally performed by applying traditional chemistry methods, imply sample preparation to separate a compound/a group of compounds and ulterior treatment to determine its concentration. Advanced methods based on electrochemical analysis, flow injection analysis, biosensors (e.g., e-tongue, e-nose), differential scanning calorimetry, gas and liquid chromatography (GC-FID, HPLC), mass spectrometry (GC–MS, LC–MS, IRMS), NMR and infrared (NIR, FT-IR, and Raman) spectroscopy have been developed during the last three decades for the detection of honey adulterants^7,10,13–16,19–24,26,27,29–33^. Each method used to detect the adulteration has its specific advantages and limitations. Traditional physicochemical analysis methods have commonly high sensitivity and selectivity. They are frequently used in the honey trade, although some of them are relatively time-consuming and require complex equipment. Non-targeted methods based on NMR spectroscopy involve high sensitivity and reproducibility, but substantial capital, operational, and maintenance costs. Techniques based on infrared spectroscopy are simple, fast, non-destructive, and relatively inexpensive, but large datasets are needed to obtain accurate results. Methods implying gas and liquid chromatography are able to detect adulteration by determining the profiles of sugars, amino acids, phenolics, and flavonoids, but they are generally time-consuming, expensive, and require skilled operators. However, there is an advanced technique, i.e., isotope ratio mass spectrometry (IRMS), that overcomes the common limitations of advanced methods and it is used as a reference technique to detect the addition of sugar syrups^13,15,19,21–24,26,29,31,32^. The ratio between stable carbon isotopes for a sample (of plant, honey, or honey extracted proteins), *R*_*sample*_ = (^13^C/^12^C)_*sample*_, is determined using a mass spectrometer and expressed depending on an internal standard (st), commonly Vienna Pee Dee Belemnite (VPDB), as *δ*^13^*C* (‰) defined by Eq. (1). A negative value of *δ*^13^*C* indicates that the sample is depleted in heavy isotope (^13^*C*), whereas a positive value shows that the sample is enriched in ^13^*C* relative to the standard. The ratio of heavy and light carbon isotopes for VPDB is *R*_*st*_ = (^13^C/^12^C)_*st*_ = 0.0112372. The values of *δ*^13^*C* for C_4_ plants (that fixate CO_2_ into 4-carbon compounds via the Hatch-Slack photosynthetic cycle) are in the range of − 20 ÷ − 10‰, whereas those for C_3_ plants (that use the Calvin–Benson–Bassham cycle to fixate CO_2_ into 3-carbon compounds) are of − 33 ÷ − 21‰^13,15,21,29,31,32^. C_3_ plants, which represent about 85% of plant species, are the main nectar source for bees. An adulteration is suspected if the difference between the value of *δ*^13^*C* for honey and that for its extracted proteins, Δ*δ*^13^*C* = *δ*^13^*C*_*H*_–*δ*^13^*C*_*P*,_ is not in the range [‒1, 1]^13,15,31^. IRMS method is useful in detecting the adulteration of honey with sugar syrups derived from C_4_-plants, e.g., sugar cane, corn, but it fails to detect the adulteration with those obtained from C_3_-plants, e.g., sugar beet. Morever, there is some concern about the precision and accuracy of this technique. IRMS method coupled with targeted physicochemical analysis methods and chemometrics (multivariate data analysis) can be a very effective alternative to discriminate between pure and adulterated honey.

1 $$ \delta^{13} C = \left( {\frac{{R_{sample} }}{{R_{st} }} - 1} \right) \times 1000. $$

Principal component analysis (PCA) and linear discriminant analysis (LDA) are dimensionality reduction techniques which are widely applied to characterize and classify food^4,8–11,13,15,16,18,22,23,25,26,30,33–36^. PCA, an unsupervised approach, aims at finding a lower dimensional space, while retaining as much variability as possible. New variables [principal components (PCs)], which are linear functions of independent variables in the original data set, are defined by determining the eigenvectors and eigenvalues of data covariance matrix. The eigenvector with the highest eigenvalue represents the direction of largest variation, the one with the second highest eigenvalue is the orthogonal direction with the next largest variation, and so forth. PCA allows visualization of data structure and identification of possible data groups. Accordingly, it is often used prior to LDA, a supervised approach which considers predefined groups^15,18,25^. Linear discriminant (LD) and classification functions are obtained by applying LDA technique to multivariate data sets. LD functions highlight the differences among the groups by maximizing the between-group variance and minimizing the within-group variance, whereas classifications functions determine to which predefined group each sample can be assigned^15,18,25,35,37^.

Romania is an important honey producer and exporter in Europe with an annual output over 20,000 t in recent years, of which about half is exported^25^. Acacia, lime, rape, sunflower, and polyfloral are the most common Romanian honey types^6,18,25,38^. This paper has aimed at evaluating the ability of physicochemical analysis methods specified in the national standard coupled with IRMS technique and chemometrics to discriminate between pure and adulterated Romanian acacia honey. Adulteration was performed directly, by mixing pure acacia honey with 3 different sugar syrups, as well as indirectly, by feeding the bees with the same syrups. In order to distinguish between pure, directly and indirectly adulterated honey, PCA and LDA were applied to experimental results in terms of moisture, ash, HMF, RS (fructose and glucose), and NRS (sucrose) contents, FA, DA, and Δ*δ*^13^*C* = *δ*^13^*C*_*H*_–*δ*^13^*C*_*P*_. There are no studies in the related literature on the detection of Romanian honey adulteration by bee-feeding based on physicochemical analysis methods and IRMS technique.

## Materials and methods

### Honey samples

The research was developed in collaboration with a beekeeper from Vâlcea county of Romania, who agreed to participate to the study with 12 hives (H1–H12), each of them containing 3 colonies of *Apis mellifera carpatica*. Three acacia honey types were prepared and analyzed, i.e., authentic or pure (P), indirectly (I) adulterated by bee-feeding with sugar syrups, and directly (D) adulterated by mixing P honey with the same syrups. Three types of industrial syrups (S1–S3 in Table 1), which are commonly employed for bee-feeding, were used for adulterating.

**Table 1:** Industrial syrup composition.

Syrup	Solid phase concentration (%)	Solid phase composition (%)				
		Fructose	Glucose	Sucrose	Maltose	Others
S1	73	39	31	30	0	0
S2	75	50	32	0	15	3
S3	82	14	43	43	0	0

Data on the experiments performed to obtain pure and adulterated honey are summarized in Table 2. P honey was produced in 3 hives (H1–H3) without bee-feeding with sugar syrups, whereas I adulterated honey was obtained in 9 hives (H4–H12) as follows: H4–H6 hives were fed with S1, H7–H9 with S2, and H10–H12 with S3. 1 L of sugar syrup was fed in each hive (H4–H12) once every 3 days, between April 1st and May 5th, 2017. Honey was collected from each hive according to Romanian standard SR 784-1^39^ and following an “average-taking” protocol^22^. D adulterated honey was prepared by mixing P honey with S1–S3 syrups (1/1 mass ratio). According to data presented in Table 2, 23 samples of honey were physicochemically analyzed. Prior to analysis, all samples were homogenized for 10 min using a mixer.

**Table 2:** Experimental runs.

No.	Honey type	Code	Description	Hives	Honey samples
1	Pure (P)	P	No bee-feeding	3 (H1–H3)	5
2	Directly (D) adulterated	D1	P honey mixed with S1		3
3		D2	P honey mixed with S2		3
4		D3	P honey mixed with S3		3
5	Indirectly (I) adulterated	I1	Bee-feeding with S1	3 (H4–H6)	3
6		I2	Bee-feeding with S2	3 (H7–H9)	3
7		I3	Bee-feeding with S3	3 (H10–H12)	3

### Physicochemical analysis

Physicochemical parameters in terms of moisture content, ash content, FA, RS content, sucrose content, DA, and HMF content were determined based on Romanian standard SR 784-3^40^. This standard was harmonized with Official methods of analysis of Association of Official Analytical Chemists (AOAC)^41^ and Harmonized methods of the European honey commission^42^.

Moisture content was measured at 20 °C with an Atago Digital Refractometer RX-5000i (Atago, USA). Ash content was evaluated as follows: a honey sample (5 g) was desiccated in a platinum dish, kept in a thermostat at 80 °C for 4 h, and further calcined at 550 °C in a laboratory furnace (Nabertherm, Germany). FA was determined by the titrimetric method, i.e.: 10 g of honey sample was dissolved in 75 mL of CO_2_-free water in a 250 mL beaker. The solution was magnetically stirred and titrated to pH 8.3 by adding 0.05 N NaOH solution. The pH was measured using a Mettler Toledo SevenMulti pH meter S40 (Mettler Toledo, Canada). RS were evaluated by reducing Soxhlet’s modification of Fehling’s solution by titration at boiling point against a solution of reducing sugars in honey in the presence of methylene blue as indicator. The difference in concentrations of invert sugar before and after the hydrolysis was multiplied by 0.95 to obtain the apparent sucrose content. DA was evaluated using a buffered solution of honey and soluble starch incubated at 40 °C. 1 mL volumes of this solution were taken at regular intervals (5 min) and their absorbance was measured at 660 nm in a Perkin Elmer Luminescence Spectrophotometer LS-50B (Perkin Elmer, UK). For HMF determination, 5 g of honey sample was mixed with 25 mL distilled water. After clarifying with Carrez reagents (I and II), the solution was diluted to 100 mL with distilled water. Absorbance of this clarified solution was measured at 284 and 336 nm in a Perkin Elmer Luminescence Spectrophotometer LS-50B (Perkin Elmer, UK) using as a reference the same solution containing 0.2% (m/v) sodium bisulphate.

The analysis of ^13^C/^12^C stable isotope ratio for honey and protein fraction extracted from honey was carried out according to the Official Methods of Analysis 998.12^43^. The values of ^13^C/^12^C ratio were determined using a Thermo Scientific FlashEA 1112 HT elemental analyzer (EA) coupled to a Delta V Continuous Flow Isotope Ratio Mass Spectrometer (CF-IRMS) (Thermo Fisher Scientific, Waltham, MA, USA) and expressed depending on PDB internal standard as *δ*^13^*C*_*H*_ and *δ*^13^*C*_*P*_. The proteins extraction involved mixing 10–12 g of honey sample with 4 mL of distilled water in a 50 mL centrifuge tube. After addition of 2 mL of 2/3 N sulphuric acid and 2 mL of 10% sodium tungstate solutions, the tube was heated to 80 °C until protein precipitated. The supernatant was removed after centrifuging and rinsing with 50 mL of distilled water. After drying in an oven (75 °C) for 6 h, the protein sample (200 μg) was placed into a tin capsule for analysis.

All reagents used for physicochemical analyzes were analytical grade and were purchased from Merck (Darmstadt, Germany).

### Statistical analysis

Univariate (one-way ANOVA) and multivariate (PCA and LDA) analyzes of physicochemical parameters were performed using Statistica 10 (StatSoft, Inc) and XLSTAT 2019.1 (Excel). A standardized data matrix with 23 rows (number of samples) and 8 columns (number of physicochemical parameters), containing autoscaled variable values, was used in PCA. In order to obtain a correct predictive classification, in LDA the samples were divided based on a selection algorithm into a training set consisting of 16 samples and a validation set containing 7 samples (one sample of each honey type, i.e., P, D1–D3, I1–I3). Accordingly, a data matrix with 16 rows and 8 columns was used to select the predictors with higher discriminative power and to build linear discriminant functions and classification functions, whereas a data matrix with 7 rows and 8 columns was used to validate the models. Raw data entered the multivariate analysis and these data were autoscaled by the software. LDA was applied using forward stepwise (FS) method (8 steps, tolerance value = 0.01, *F to enter* value = 1, *F to remove* value = 0) and considering P, D, and I honey as predefined groups. In FS setting, the variables entered into the discriminant function model one by one and only the variables which contributed significantly to the discrimination between groups (with low levels of partial Wilks’ Lambda and large *F* values, respectively) were selected. At each step, the multiple correlation (*R*^2^) for each variable with all other variables included in the model was calculated. Tolerance value of a variable, which is a measure of its redundancy, was determined as 1 − *R*^2^.

## Results and discussions

### Experimental results

Table 3 contains mean, standard deviation, and variation range of characteristic physicochemical parameters of pure and adulterated honey (23 samples). Characteristic value of moisture, ash, RS, sucrose, and HMF contents, FA, and DA were compared with limit levels specified in the national standard SR 784-3^40^, whereas the values of *δ*^13^*C*_*H*_, *δ*^13^*C*_*P*_, and Δ*δ*^13^*C* = *δ*^13^*C*_*H*_‒*δ*^13^*C*_*P*_ were compared with those reported in the related literature^15,19,21,23,24,26,29,31,32^.

**Table 3:** Limit level, mean, standard deviation, and range (min–max) for characteristic physicochemicals parameters of honey samples.

No.	Parameter	Limit	Sample						
			P	I1	I2	I3	D1	D2	D3
1	Moisture content (%)	Max. 20	16.8 ± 0.36 16.3–17.2	22.2 ± 0.35 21.9–22.6	18.1 ± 0.17 18.0–18.3	16.1 ± 0.33 15.9–16.5	20.8 ± 0.28 20.6–21.1	19.7 ± 0.30 19.5–20.1	17.1 ± 0.13 17.0–17.2
2	Ash content (% × 10^3^)	Max. 500	8.1 ± 0.13 8.0–8.3	10 ± 0.10 10.0–10.2	20 ± 0.15 20.1–20.4	4 ± 0.03 4.00–4.05	2 ± 0.02 2.01–2.05	4 ± 0.03 4.01–4.07	8 ± 0.02 7.98–8.02
3	FA (meq/kg)	Max. 40	0.75 ± 0.02 0.72–0.77	6.50 ± 0.10 6.4–6.6	4.03 ± 0.12 3.9–4.1	6.03 ± 0.21 5.8–6.2	5.00 ± 0.20 4.8–5.2	5.03 ± 0.06 5.0–5.1	5.03 ± 0.12 4.9–5.1
4	RS content (%)	Min. 70	70.5 ± 1.48 68.7–72.5	66.5 ± 0.81 65.7–67.3	65.3 ± 0.58 64.8–66.0	58.1 ± 0.48 57.5–58.5	66.0 ± 1.05 65.0–67.1	66.5 ± 0.68 65.8–67.2	65.3 ± 0.61 64.8–66.0
5	Sucrose content (%)	Max. 5	3.79 ± 0.04 3.75–3.85	14.7 ± 0.22 14.5–15.0	6.81 ± 0.05 6.76–6.85	7.60 ± 0.08 7.51–7.67	6.17 ± 0.09 6.08–6.25	6.65 ± 0.08 6.59–6.74	6.33 ± 0.06 6.28–6.39
6	DA (Gothe units/g)	Min. 6.5	13.9 ± 0.25 13.6–14.2	5.01 ± 0.01 5.00–5.02	5.04 ± 0.03 5.01–5.07	5.02 ± 0.09 4.94–5.11	6.41 ± 0.11 6.30–6.51	6.44 ± 0.08 6.38–6.53	6.43 ± 0.10 6.30–6.50
7	HMF content (mg/kg)	Max. 15	1.21 ± 0.03 1.18–1.25	23.0 ± 0.13 22.9–23.2	40.9 ± 0.38 40.6–41.3	25.7 ± 0.31 25.4–26.0	18.5 ± 0.18 18.3–18.7	24.9 ± 0.29 24.6–25.2	20.2 ± 0.20 20.0–20.4
8	│ *δ*^13^*C*_*H*_ │ (‰)		23.7 ± 0.59 23.0–24.4	26.3 ± 0.20 26.1–26.5	12.4 ± 0.12 12.3–12.5	14.9 ± 0.15 14.7–15.0	24.5 ± 0.50 24.0–25.0	18.6 ± 0.55 18.0–19.1	17.2 ± 0.25 17.0–17.5
9	│ *δ*^13^*C*_*P*_ │ (‰)		24.1 ± 0.52 23.5–24.8	29.7 ± 0.25 29.5–30.0	23.5 ± 0.45 23.0–23.9	20.3 ± 0.70 19.6–21.0	27.6 ± 0.65 27.0–28.3	25.2 ± 0.75 24.5–26.0	25.2 ± 0.80 24.4–26.0
10	Δ*δ*^13^*C *(‰)	‒1 ÷ 1	0.46 ± 0.09 0.4–0.6	3.43 ± 0.06 3.4–3.5	11.1 ± 0.46 10.7–11.6	5.43 ± 0.55 4.9–6.0	3.13 ± 0.15 3.0–3.3	6.67 ± 0.21 6.5–6.9	7.97 ± 0.55 7.4–8.5

Moisture content is a relevant parameter as it affects the viscosity, density, taste, flavour, colour, crystallization, and fermentation of honey^2,5,17,32^. A high water content can accelerate the crystallization as well as produce fermentation during the storage^2,6,12,17,32^. It is observed that only I1 and D1 samples contain more moisture than the regulated limit (max. 20%). The mean values of moisture content for I (18.8%) and D (19.2%) samples are similar and higher than the mean value of P samples (16.8%).

Ash or mineral content is an indicator affecting the colour and flavour of honey. Usually, the higher the ash content, the darker the colour and the stronger the flavour^5^. The mean values of ash content for P (0.0081%), I (0.0113%), and D (0.0047%) samples are much lower than the imposed limit (max. 0.5%). There were no differences in colour and flavour among the samples.

FA is mainly due to the presence of organic acids in equilibrium with lactones or internal esters and inorganic ions, e.g., chloride, sulphate, phosphate, and it can heavily influence the honey taste^2,5,7,17,28^. An increase in FA can occur over time as effect of acid formation (e.g., gluconic acid from glucose, formic and levulinic acids from HMF) as well as in the case of fermentation (by producing acetic acid from ethylic alcohol resulted in the fermentation process in the presence of honey yeasts)^5,12^. All samples have values of FA much lower than the regulated limit (max. 40 meq/kg). The mean values of FA for I (5.5 meq/kg) and D (5.0 meq/kg) samples are similar and about 7 times higher than the mean value of P samples (0.75 meq/kg).

All adulterated samples have values of sucrose content higher than the regulated limit (max. 5%). The mean value of sucrose content for I samples (9.71%) is higher than those of D and P samples (6.38% and 3.79%). On the other hand, all adulterated samples have values of RS (fructose and glucose) content lower than the imposed limit (min. 70%). The mean value of RS content for I samples (63.3%) is lower than those of D and P samples (65.9% and 70.5%). Accordingly, the honey samples adulterated by bee-feeding contain more sucrose which has not been converted to fructose and glucose.

DA and HMF content are indicators of honey freshness. A high quality honey is characterized by high values of DA and low level of HMF. In the case of heating or storage for a long time, DA decreases and HMF content increases (HMF can be produced by fructose and glucose decomposition)^5,12,26,32^. Moreover, a higher value of HMF content can indicate an adulteration with inverted sugar syrup, because HMF can be formed by heating sucrose solutions to obtain inverted syrup, whereas a low level of DA can be an effect of an indirect adulteration^5^. All adulterated samples have values of HMF content higher and of DA lower than the regulated limits (max. 15 mg/kg and min. 6.5 Gothe units/g). The mean value of HMF content for I samples (29.9 mg/kg) is higher than those of D and P samples (21.2 mg/kg and 1.21 mg/kg), whereas the mean value of DA for I samples (5 Gothe units/g) is lower than those of D and P samples (6.5 Gothe units/g and 13.9 Gothe units/g).

The values of *δ*^13^C_*H*_ and *δ*^13^*C*_*P*_ (− 24.4 ÷ − 23.0 and − 24.8 ÷ − 23.5 for P, − 26.5 ÷ − 12.3 and − 30.0 ÷ − 19.6 for I, − 25.0 ÷ − 17.0 and − 28.3 ÷ − 24.4 for D honey) are within the ranges reported by other researchers^15,19,21,23,24,26,29,31,32^. Levels of Δ*δ*^13^C = *δ*^13^C_*H*_–*δ*^13^*C*_*P*_ for I and D samples (3.4 ÷ 11.6 and 3.0 ÷ 8.5) are more than 5 times higher than those for P samples (0.4 ÷ 0.6). These results indicate that P honey is pure (values of Δ*δ*^13^*C* are in the range [− 1, 1]), whereas I and D samples are adulterated.

The data summarized in Table 3 highlight that some physicochemical parameters of adulterated honey are within the limit levels specified in the national standard. Consequently, only checking these parameters is not sufficient to detect the adulteration. Physicochemical analysis methods coupled with chemometrics could be an effective alternative to discriminate between pure and adulterated honey.

### Statistical analysis

One-way ANOVA was used to determine the effect of honey type, i.e., pure (P), directly adulterated (D1–D3), and indirectly adulterated (I1-I3), on the physicochemical parameters considered in the experimental research. Data summarized in Supplementary Table S1 (*F*_*cr*_ = 2.741, *F* ≥ 52.5, and *p *value ≤ 1.2 × 10^–9^) reveal that the influence of honey type on all physicochemical parameters is statistically significant.

PCA and LDA were applied to discriminate between pure and adulterated honey samples based on their physicochemical parameters (independent variables or predictors). Eight independent variables were considered in the multivariate analysis, i.e., moisture, ash, RS, sucrose, and HMF contents, FA, DA, and Δ*δ*^13^*C*.

PCA results referring to eigenvalues, explained variance of principal components (PCs), and factor (PC) coordinates of variables are presented in Supplementary Tables S2 and S3. Tabulated data highlight that the eigenvalues corresponding to PC1 (4.32), PC2 (1.78), and PC3 (1.32) are greater than 1 and these first three PCs explain 92.8% (54.0% + 22.3% + 16.5%) of the total variance. Depending on their coordinates in the first three PCs (factor loadings), the most important variables are as follows: (i) DA (0.99), HMF content (− 0.91), FA (− 0.91), Δ*δ*^13^*C* (*l*_*v*_ = − 0.73), RS content (0.69), and sucrose content (− 0.68) for PC1, (ii) moisture content (0.71) for PC2, and (iii) ash content (0.70) for PC3.

**Figure 1:**
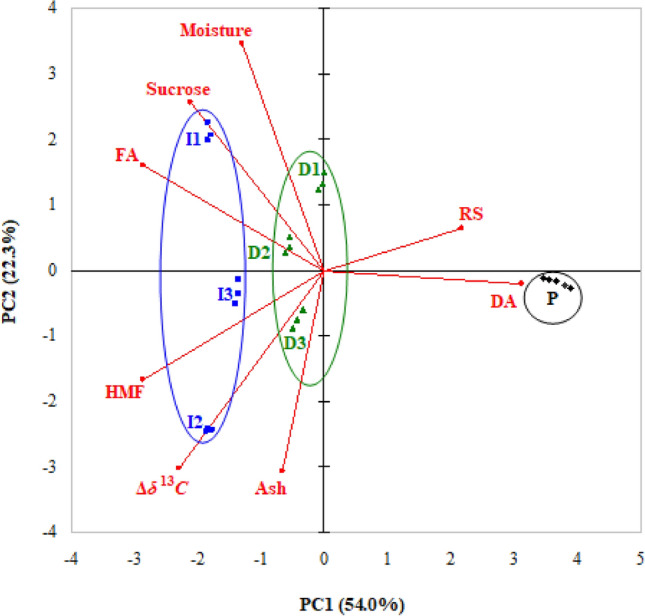
PCA bi-plot (projections of samples and variables on the factor-plane PC1–PC2).

Only PC1 and PC2 were retained in the analysis, because the cumulative percentage of total variance explained by them (76.3%) was higher than 70%^16^. Projections of variables and cases (honey samples) on the factor-plane PC1–PC2 are shown in Fig. 1. Based on the sign and magnitude of factor loadings, PC1 can discriminate between pure honey samples with high values of DA and RS content along with low levels of FA, Δ*δ*^13^*C*, HMF and sucrose contents and adulterated samples. Projections of cases on the factor-plane PC1–PC2 highlight a good discrimination between P, D, and I honey groups on the PC1 direction. PC1 coordinates of P samples (3.5 ÷ 3.9) correspond to higher values of DA (13.6 ÷ 14.2 Gothe units/g) and RS content (68.7 ÷ 72.5%) along with lower levels of FA (0.75 ÷ 0.77 meq/kg), Δ*δ*^13^*C* (0.4 ÷ 0.6‰), HMF content (1.18 ÷ 1.25 mg/kg), and sucrose content (3.75 ÷ 3.85%). Concurrently, PC1 coordinates of D1–D3 samples (− 0.6 ÷ 0) and I1–I3 samples (− 1.9 ÷ − 1.4) correspond to lower values of DA (6.30 ÷ 6.53 and 4.94 ÷ 5.11 Gothe units/g) and RS content (64.8 ÷ 67.2 and 57.5 ÷ 67.3%) as well as to higher levels of FA (4.8 ÷ 5.2 and 3.9 ÷ 6.6 meq/kg), Δ*δ*^13^*C* (3 ÷ 8.5 and 3.4 ÷ 11.6‰), HMF content (18.3 ÷ 25.2 and 22.9 ÷ 41.3 mg/kg), and sucrose content (6.08 ÷ 6.74 and 6.76 ÷ 15%). On the other hand, PC2 can discriminate between the samples adulterated with S1 syrup (with PC2 coordinates of 2.1 ÷ 2.3 for I1 and 1.2 ÷ 1.5 for D1 samples), having high values of moisture content (21.9 ÷ 22.6% and 20.6 ÷ 21.1%, respectively) and those adulterated with S2 and S3 syrups (with PC2 coordinates of − 2.5 ÷ 0.5), which had lower levels of moisture content (15.9 ÷ 20.1%). Accordingly, PC1 is mostly related to the adulteration and PC2 to the type of syrup used to adulterate the honey. It appears in the bi-plot (Fig. 1) that the separation on PC1 direction is comparable in magnitude to that on PC2 direction. However, it must be considered that PC1 accounts for 54.0% of the total variance, while PC2 only 22.3%.

LDA was applied to obtain the variables with higher discriminative power and their corresponding discriminant and classification functions. The most important discriminative physicochemical parameters determined by FS method were DA, HMF, sucrose, and RS contents. Accordingly, only these parameters were further taken into account to determine discriminant and classification functions. The first linear discriminant (LD1) function explains 98.7% of the discriminative power, whereas the second (LD2) accounts for 1.3%, the eigenvalues corresponding to LD1 and LD2 functions being of 785.23 and 10.04, respectively. Values of standardized linear discriminant function coefficients and factor structure coefficients (factor loadings) are summarized in Table 4. Standardized discriminant function coefficients represent partial (corrected for the other predictors) contribution of each predictor to the discriminant function score, whereas structure coefficients denote the simple correlations (not corrected for the other predictors) between predictors and discriminant functions scores. Tabulated data on standardized coefficients highlight that LD1 function is negatively correlated with RS content, positively correlated with DA, sucrose and HMF contents, and DA has the most significant effect. On the contrary, LD2 function is positively correlated with RS content, negatively correlated with DA, sucrose and HMF contents, and DA has an insignificant effect. Both types of coefficients indicate that DA and sucrose content had the largest contribution to the discrimination by LD1 and LD2, respectively.

**Table 4:** LDA results in terms of standardized discriminant function coefficients and factor structure coefficients.

No.	Variable name	Standardized discriminant function coefficients		Factor structure coefficients	
		LD1	LD2	LD1	LD2
1	RS content	− 0.947	2.410	0.716	0.220
2	Sucrose content	0.810	− 2.989	− 0.631	− 0.384
3	DA	1.215	− 0.428	0.999	0.033
4	HMF content	0.558	− 2.598	− 0.891	− 0.194

**Figure 2:**
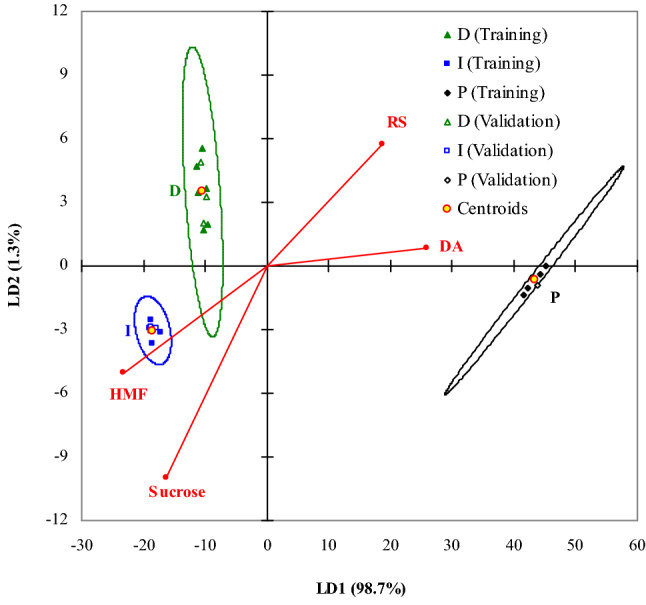
LDA bi-plot (projections of samples and the most important discriminative variables on the factor-plane LD1–LD2).

Projections of training honey samples on the factor-plane LD1–LD2 are presented in Fig. 2. P honey samples are plotted much further to the right in the scatterplot than D and I ones, LD1 scores being as follows: 41.58 ÷ 45.12 for P, − 11.44 ÷ − 9.49 for D, and − 19.03 ÷ − 17.22 for I samples. Accordingly, LD1 function separates all three honey classes (groups). Taking into account the factor loadings represented in LDA bi-plot (Fig. 2), LD1 function mostly discriminates between pure and adulterated honey, the most discriminative predictor being DA (13.6 ÷ 14.2, 6.30 ÷ 6.53, and 4.94 ÷ 5.11 Gothe units/g for P, D, and I honey classes). On the other hand, LD2 function discriminates between D and I honey types (LD2 scores of 1.67 ÷ 5.54 for D and − 3.63 ÷ − 2.56 for I samples), the most discriminative predictor being sucrose content (6.08 ÷ 6.74% for D and 6.76 ÷ 15% for I). LD1 function accounts for 98.7% of the discriminative power, consequently the most significant discrimination is possible for all three honey types by this function. Mean values of factor scores for each group (centroids) and 95% confidence ellipses for training set as well as projections of validation honey samples on the factor-plane LD1–LD2 are also shown in Fig. 2. It is observed that all points corresponding to LD1 and LD2 scores for validation set are included in the ellipses corresponding to their group.

Honey samples (cases) were classified into the group to which they were closest. Mahalanobis distances between each case and the group centroids were calculated. According to Bayes rule, the probability that a case belongs to a group, i.e., posterior classification probability (*PP*), was determined depending on squared Mahalanobis distance (*SMD*) and a priori classification probability (*APP*). *APP* values were assumed to be proportional to the number of samples in each group (*APP*_*P*_ = 0.25, *APP*_*D*_ = 0.375, and *APP*_*I*_ = 0.375). A case can be classified based on largest value of *PP*. Prior and posterior classification and levels of *SMD* and *PP* for training (T) and validation (V) sets are summarized in Supplementary Table S4.

Moreover, linear classification functions based on linear discriminant functions were determined for each honey group. Table 5 contains characteristic coefficients of classification functions, i.e., *c*_*ij*_ and *c*_*j*_, where *i* and *j* denote independent variable and group, respectively. These linear classification functions can be used directly to classify cases, i.e., a case is assigned to the group for which it has the highest value of classification function score (*CFS*). Values of *CFS* determined from raw data for both T and V sets are also summarized in Supplementary Table S4. Classification (confusion) matrices presented in Supplementary Table S5 reveal that 100% of honey samples were correctly assigned to their original group for both T and V sets.

**Table 5:** LDA results in terms of characteristic coefficients of classification functions.

	P	D	I
RS content	− 8.08	13.71	10.79
Sucrose content	8.60	− 14.40	− 9.05
DA	620.55	189.98	145.39
HMF content	1.71	− 5.49	− 3.29
*c*_*j*_	− 4041.17	− 958.86	− 614.72

## Conclusions

The paper aimed at measuring various physicochemical parameters of honey samples and discriminating the pure and adulterated honey based on these parameters using PCA and LDA as chemometric tools. Acacia pure (P) and indirectly (I) adulterated honey, obtained by bee-feeding with 3 different industrial sugar syrups, were produced in a small apiary in the Vâlcea county of Romania, whereas directly (D) adulterated honey was prepared by mixing P honey with the same syrups. Honey samples were analysed in terms of moisture, ash, HMF, RS (glucose and glucose), and sucrose contents, FA, DA, *δ*^13^C_*H*_, and *δ*^13^*C*_*P*_.

Adulteration led to an increase in water content (by about 10%), FA (about 7 times), sucrose content (2.6 and 1.7 times for I and D samples, respectively), HMF content (about 25 and 18 times for I and D samples, respectively), and Δ*δ*^13^*C* = *δ*^13^*C*_*H*_‒*δ*^13^*C*_*P*_ (about 17 and 15 times for I and D samples, respectively) as well a decrease in RS content (by about 10%) and DA (2.8 and 2.1 times for I and D samples, respectively) than the mean value of P samples. For the types and dosage of sugar syrups used in this study, indirect adulteration had effects similar to those produced by direct adulteration. The values of DA, HMF, sucrose, and RS contents for D and I honey samples were not within the ranges imposed by the national standard.

Moisture, ash, RS, sucrose, and HMF contents, FA, DA, and Δ*δ*^13^*C*, were the physicochemical parameters considered in the multivariate analysis. According to PCA, the 8-dimensional feature space was reduced to a 2-dimensional one, where the directions of new axes were defined by PC1 and PC2 eigenvectors. PC1, explaining 54.0% of total variance, was dominated by DA, HMF content, FA, Δ*δ*^13^*C*, RS and sucrose contents, whereas PC2, accounting for 22.3% of total variance, was dominated by moisture content. Three well separated honey groups (P, D, and I) on the PC1 direction were obtained by projecting the honey samples on the factor-plane PC1–PC2. Starting from these predefined groups, forward stepwise LDA revealed that the most important discriminative physicochemical parameters were DA, HMF, sucrose, and RS contents. Linear discriminant functions and classification functions were built based on a training dataset and then these models were validated using a validation dataset. Cases were classified based on the largest values of posterior classification probability and classification function score. Characteristic confusion matrices of both training and validation sets indicated that 100% of honey samples were correctly assigned to their original group. Accordingly, the evaluation of physicochemical parameters using PCA and LDA was very effective to discriminate between pure, directly and indirectly adulterated honey. Based on the physicochemical parameters in terms of DA, HMF, sucrose, and RS contents, any sample of acacia honey could be simply and quickly classified as pure, directly or indirectly adulterated by means of classification functions obtained by applying LDA. However, the study has some limitations, e.g., a small number of acacia honey samples, a relative similarity of them (all coming from the same producer and being produced within the same few months). The analysis could be extended using numerous samples of different types of honey collected from several producers over a longer period.

## Supplementary information

Supplementary Information.
