# Supplementary material for: Characterization and classification of Romanian acacia honey based on its physicochemical parameters and chemometrics
Source: Sci Rep. 2020 Nov 26;10:20690. doi: 10.1038/s41598-020-77685-9 (PMC7691509; doi:10.1038/s41598-020-77685-9)
Supplement: Supplementary file 1 — Supplementary Information. [file 41598_2020_77685_MOESM1_ESM.doc]

**Characterization and classification of Romanian acacia honey based on its physicochemical parameters and chemometrics**

(Supplementary information)

**Mihaela Emanuela Crăciun^1^, Oana Cristina Pârvulescu^2*^, Andreea Cristina Donise^2^, Tănase Dobre^2^, Dumitru Radu Stanciu^3^**

^1^University POLITEHNICA of Bucharest, Department of Analytical Chemistry and Environmental Engineering, 1-6 Gheorghe Polizu, 011061, Bucharest, Romania

^2^University POLITEHNICA of Bucharest, Department of Chemical and Biochemical Engineering, 1-6 Gheorghe Polizu, 011061, Bucharest, Romania

^3^University POLITEHNICA of Bucharest, Department of Economic Engineering, 313 Splaiul Independentei, 060042, Bucharest, Romania

**^*^**Corresponding author. E-mail address: oana.parvulescu@yahoo.com.

**Supplementary (S) tables**

| No. | Parameter | *F_cr_* | *F* | *p*-value |
| --- | --- | --- | --- | --- |
| 1 | Moisture content (%) | 2.741 | 189.57 | 6.0E-14 |
| 2 | Ash content (%) |  | 12386 | 2.1E-28 |
| 3 | FA (meq/kg) |  | 983.36 | 1.3E-19 |
| 4 | RS content (%) |  | 52.541 | 1.2E-09 |
| 5 | Sucrose content (%) |  | 3804.2 | 2.6E-24 |
| 6 | DA (Gothe units/g) |  | 2245.9 | 1.8E-22 |
| 7 | HMF content (mg/kg) |  | 10455 | 8.0E-28 |
| 8 | │ *δ*^13^*C_H_* │ (‰) |  | 509.87 | 2.4E-17 |
| 9 | │ *δ*^13^*C_P_* │(‰) |  | 75.246 | 8.0E-11 |
| 10 | Δ*δ*^13^*C*=*δ*^13^*C_H_*‒*δ*^13^*C_P_* (‰) |  | 395.56 | 1.8E-16 |

**Table S1.** One-way ANOVA results for pure and adulterated honey samples.

| Principal component | Eigenvalue | | % Total variance | |
| --- | --- | --- | --- | --- |
|  | PC | Cumulative | PC | Cumulative |
| PC1 | 4.320 | 4.320 | 54.00 | 54.00 |
| PC2 | 1.782 | 6.102 | 22.28 | 76.28 |
| PC3 | 1.322 | 7.424 | 16.52 | 92.80 |
| PC4 | 0.428 | 7.852 | 5.354 | 98.15 |
| PC5 | 0.115 | 7.967 | 1.432 | 99.59 |
| PC6 | 0.018 | 7.985 | 0.230 | 99.82 |
| PC7 | 0.014 | 7.999 | 0.169 | 99.99 |
| PC8 | 0.001 | 8.000 | 0.015 | 100.0 |

**Table S2.** PCA results in terms of eigenvalues and explained variance for pure and adulterated honey samples.

| No. | Variable name | PC1 | PC2 | PC3 | PC4 | PC5 | PC6 | PC7 | PC8 |
| --- | --- | --- | --- | --- | --- | --- | --- | --- | --- |
| 1 | Moisture content | ‒0.411 | **0.706** | 0.487 | 0.272 | 0.139 | 0.036 | ‒0.028 | ‒0.006 |
| 2 | Ash content | ‒0.210 | ‒0.625 | **0.701** | ‒0.259 | 0.063 | 0.044 | 0.030 | ‒0.006 |
| 3 | FA | **‒0.914** | 0.325 | ‒0.201 | 0.008 | ‒0.124 | 0.007 | 0.057 | ‒0.020 |
| 4 | RS content | **0.689** | 0.131 | 0.651 | 0.234 | ‒0.164 | ‒0.035 | 0.037 | 0.006 |
| 5 | Sucrose content | **‒0.676** | 0.522 | 0.317 | ‒0.396 | ‒0.101 | ‒0.032 | ‒0.040 | 0.007 |
| 6 | DA | **0.989** | ‒0.042 | 0.093 | ‒0.087 | ‒0.004 | ‒0.043 | ‒0.041 | ‒0.025 |
| 7 | HMF content | **‒0.911** | ‒0.341 | 0.117 | 0.120 | 0.119 | ‒0.102 | 0.010 | ‒0.001 |
| 8 | Δ*δ*^13^*C* | **‒0.729** | ‒0.616 | 0.077 | 0.232 | ‒0.157 | 0.024 | ‒0.062 | ‒0.003 |

**Table S3.** PCA results in terms of factor coordinates of variables for pure and adulterated honey samples.

| No. | Set | Classification | | Squared Mahalanobis distance | | | Posterior probability | | | Classification function score | | |
| --- | --- | --- | --- | --- | --- | --- | --- | --- | --- | --- | --- | --- |
|  |  | Prior | Posterior | *SMD_P_* | *SMD_I_* | *SMD_D_* | *PP_P_* | *PP_I_* | *PP_D_* | *CFS_P_* | *CFS_I_* | *CFS_D_* |
| 1 | T | P | P | 4.01 | 3698.8 | 2804.6 | **1** | 0 | 0 | **3938.8** | 2091.3 | 2538.9 |
| 2 |  | P | P | 6.60 | 3608.8 | 2730.8 | **1** | 0 | 0 | **3864.7** | 2063.6 | 2503.0 |
| 3 |  | P | P | 6.73 | 4052.8 | 3100.2 | **1** | 0 | 0 | **4214.3** | 2191.1 | 2667.9 |
| 4 |  | P | P | 3.94 | 3951.2 | 3016.1 | **1** | 0 | 0 | **4134.7** | 2161.0 | 2629.0 |
| 5 |  | I | I | 3843.5 | 5.96 | 122.10 | 0 | **1** | 0 | -1304.8 | **613.8** | 556.2 |
| 6 |  | I | I | 3868.6 | 7.00 | 117.68 | 0 | **1** | 0 | -1301.3 | **629.4** | 574.5 |
| 7 |  | I | I | 3900.5 | 5.84 | 120.54 | 0 | **1** | 0 | -1328.6 | **618.6** | 561.8 |
| 8 |  | I | I | 3876.5 | 6.78 | 113.77 | 0 | **1** | 0 | -1298.5 | **636.3** | 583.3 |
| 9 |  | I | I | 3816.3 | 6.34 | 111.19 | 0 | **1** | 0 | -1332.4 | **572.6** | 520.5 |
| 10 |  | I | I | 3678.4 | 6.67 | 95.38 | 0 | **1** | 0 | -1231.9 | **603.8** | 559.9 |
| 11 |  | D | D | 3031.9 | 111.21 | 4.69 | 0 | 0 | **1** | -573.1 | 887.1 | **940.8** |
| 12 |  | D | D | 2930.7 | 140.26 | 6.24 | 0 | 0 | **1** | -457.6 | 937.5 | **1004.9** |
| 13 |  | D | D | 2885.9 | 90.68 | 5.42 | 0 | 0 | **1** | -515.1 | 882.4 | **925.5** |
| 14 |  | D | D | 2800.9 | 108.02 | 5.62 | 0 | 0 | **1** | -430.7 | 915.6 | **967.3** |
| 15 |  | D | D | 2977.1 | 98.89 | 2.53 | 0 | 0 | **1** | -560.4 | 878.6 | **927.2** |
| 16 |  | D | D | 2855.7 | 119.79 | 2.27 | 0 | 0 | **1** | -450.8 | 917.1 | **976.3** |
| 17 | V | P | P | 3.06 | 3883.8 | 2964.1 | **1** | 0 | 0 | **4074.6** | 2134.1 | 2594.4 |
| 18 |  | I | I | 3864.8 | 6.25 | 117.69 | 0 | **1** | 0 | -1305.1 | **624.1** | 568.8 |
| 19 |  | I | I | 3880.9 | 5.91 | 117.44 | 0 | **1** | 0 | -1311.9 | **625.5** | 570.2 |
| 20 |  | I | I | 3758.3 | 5.84 | 101.98 | 0 | **1** | 0 | -1286.7 | **589.5** | 541.8 |
| 21 |  | D | D | 2950.3 | 125.56 | 4.08 | 0 | 0 | **1** | -498.3 | 914.0 | **975.2** |
| 22 |  | D | D | 2890.0 | 93.57 | 4.47 | 0 | 0 | **1** | -507.1 | 891.0 | **936.0** |
| 23 |  | D | D | 2849.3 | 115.78 | 2.39 | 0 | 0 | **1** | -458.6 | 908.1 | **965.2** |

**Table S4.** LDA results in terms of squared Mahalanobis distance, posterior classification probability, and classification function score for training (T) and validation (V) sets.

| Set | Group |  | P | D | I | Total | % Correct | Set | Group | P | D | I | Total | % Correct |
| --- | --- | --- | --- | --- | --- | --- | --- | --- | --- | --- | --- | --- | --- | --- |
| T | P |  | 4 | 0 | 0 | 4 | 100 | V | P | 1 | 0 | 0 | 1 | 100 |
|  | D |  | 0 | 6 | 0 | 6 | 100 |  | D | 0 | 3 | 0 | 3 | 100 |
|  | I |  | 0 | 0 | 6 | 6 | 100 |  | I | 0 | 0 | 3 | 3 | 100 |
|  | Total |  | 4 | 6 | 6 | 16 | 100 |  | Total | 1 | 3 | 3 | 7 | 100 |

**Table S5.** Classification (confusion) matrices for training (T) and validation (V) sets.
